# A Randomized-Controlled Trial Examining Telephone-Based Cognitive Behavioral Therapy for Patients After Metabolic and Bariatric Surgery: 18 Month Follow-up Results

**DOI:** 10.1007/s11695-025-08163-2

**Published:** 2025-09-01

**Authors:** Stephanie E. Cassin, Katey E. Park, Samantha E. Leung, Clement Ma, George Tomlinson, Raed Hawa, Susan Wnuk, Timothy Jackson, David Urbach, Allan Okrainec, Jennifer Brown, Daniella Sandre, Sanjeev Sockalingam

**Affiliations:** 1 Toronto Metropolitan University, Toronto, Canada; 2 University Health Network, Toronto, Canada; 3 University of Toronto, Toronto, Canada; 4 Centre for Addiction and Mental Health, Toronto, Canada; 5 Women’s College Hospital, Toronto, Canada; 6 Ottawa Hospital, Ottawa, Canada

**Keywords:** Metabolic and bariatric surgery (MBS), Cognitive-behavioral therapy (CBT), Psychosocial support, Mental health, Pathological eating

## Abstract

**Background:**

Telephone-based Cognitive Behavioral Therapy (Tele-CBT) has shown to reduce disordered eating and psychological distress after metabolic and bariatric surgery (MBS). However, it is currently unknown how Tele-CBT impacts outcomes long term, and if differences in weight loss trajectories following Tele-CBT emerge with a long-term follow-up. This study aimed to identify whether Tele-CBT remains effective at 18 months post-intervention for improving psychological distress and maladaptive eating, and mitigating recurrent weight gain.

**Methods:**

This large, multisite randomized control trial was conducted at three hospital-based MBS programs in Ontario, Canada. Participants (*n* = 306) were randomized 1:1 to receive either Tele-CBT or standard MBS care. The primary outcome was percentage of total weight loss (%TWL). Secondary outcomes included disordered eating (Binge Eating Scale, Emotional Eating Scale) and psychological distress (Patient Health Questionnaire-9, Generalized Anxiety Disorder-7). Linear mixed models assessed group-by-time interactions across five time points: baseline (1-year post-MBS), post-intervention, and 3-, 12-, and 18-month follow-ups.

**Results:**

Tele-CBT resulted in significant post-intervention improvements in binge eating (*MD* = − 0.46, *p* < .001), emotional eating (*MD* = − 0.14, *p* = 0.01), anxiety (*MD* = − 0.40, *p* < .001), and depressive symptoms (*MD* = − 0.47, *p* < .001). These improvements were all sustained at 3 months post-intervention (*p* < .05) whereas only improvements for emotional eating were sustained at 12 months post-intervention (*MD* = − 0.15, *p* = 0.01). There were no significant differences in %TWL trajectories between the Tele-CBT and control groups.

**Conclusions:**

Tele-CBT provides psychological benefits, particularly in reducing emotional eating. Findings highlight the need for continued psychosocial support to sustain other psychological benefits and mitigate recurrent weight gain post-MBS and further research on optimizing intervention timing and duration.

**Trial Registration:**

**ClinicalTrials.gov Identifier:** NCT03315247.

**Supplementary Information:**

The online version contains supplementary material available at 10.1007/s11695-025-08163-2.

## Introduction

Metabolic and bariatric surgery (MBS) is an effective intervention for severe obesity, leading to significant weight loss, improved health-related quality of life (HRQOL), and reduction of obesity-related comorbidities [1–3]. However, long-term weight maintenance after surgery remains a challenge, with substantial variability in patients’ weight loss trajectories and HRQOL outcomes [4, 5]. Moreover, data from the Longitudinal Assessment of Bariatric Surgery-2 (LABS-2) prospective cohort showed that 67% of patients experienced recurrent weight gain of ≥ 20% of their maximum weight loss at 5 years post-surgery [6].

Recurrent weight gain or suboptimal clinical response following MBS result from multiple factors, including psychological factors [7, 8]. One key psychological factor is the persistence of maladaptive eating behaviors such as binge eating, emotional eating, and loss-of-control eating, which are strongly associated with suboptimal weight outcomes and psychological distress [8, 9]. In addition, depression post-MBS can occur in approximately 15% of patients and has been associated with disordered eating and suboptimal weight loss [10]. Therefore, while MBS has durable treatment outcomes for obesity, many patients experience disordered eating behaviors and psychological distress that interfere with weight maintenance and can negatively impact HRQOL outcomes [4, 11].

Cognitive behavioral therapy (CBT), a well-established intervention for treating psychiatric conditions such as depression, anxiety, and disordered eating, has demonstrated efficacy in improving disordered eating in patients before and after MBS [12, 13]. However, evidence suggests that CBT interventions are more beneficial post-MBS than before surgery, aligning with patients’ preferences for CBT support beyond the first year after MBS [12]. Despite this evidence, access to in-person CBT remains a challenge for many patients, as geographical, logistical, and mobility issues can limit appointment attendance.

To address this gap, virtual interventions, such as telephone-based CBT (Tele-CBT), offer a promising alternative by providing remote psychological support to post-MBS patients. In a series of clinical trials, Tele-CBT has demonstrated moderate to large effect sizes in terms of improving disordered eating, depressive symptoms, and anxiety [13–16]. Published in 2023, the largest RCT to date examining the effect of a psychosocial intervention on weight loss, disordered eating, and psychological distress post-MBS (*n* = 306) demonstrated significantly greater reductions in binge eating, emotional eating, depressive, and anxiety symptoms in patients receiving Tele-CBT compared to standard care (routine psychosocial follow-up appointments) that were sustained at 3 months post-Tele-CBT intervention [15]. However, weight outcomes were not significantly different between the Tele-CBT group and standard care. These findings reinforced the need for longer-term studies to examine the durability of Tele-CBT effects on these outcomes post-MBS and to identify if differences in weight loss trajectories emerge with a longer follow-up period.

The current study delivered the Tele-CBT intervention at 12 months post-operatively. Although many patients experience improvements in disordered eating and psychological functioning post-surgery, prior research suggests that the 12-month mark may be an ideal time for psychosocial interventions. In a qualitative study conducted by Santiago and colleagues, patients identified 12 months post-surgery as the most appropriate time for CBT, describing this period as when old habits begin to resurface and motivation declines [17]. Consistent with this, a systematic review concluded that psychosocial interventions are most effective when delivered *before* significant problematic eating behaviours and weight recurrence [12]. In a large prospective study (*n* = 844), Nasirzadeh et al. found that binge eating, emotional eating, and loss of control over eating increased between 1 and 3 years post-surgery. Notably, binge eating at 12 months post-operatively significantly predicted lower %TWL at 24 months [9]. Thus, Tele-CBT was delivered 12 months post-MBS to equip patients with coping skills and behavioral strategies before disordered eating patterns re-emerge or become more difficult to manage.

The current follow-up study examines the long-term effects of Tele-CBT on weight loss, disordered eating, and psychological distress at 3 years post-MBS. Building on the short-term findings, this study evaluates whether the psychosocial benefits of Tele-CBT observed at 3 months, specifically significant improvements in disordered eating, depressive, and anxiety symptoms, persist over time and contribute to better weight loss outcomes. Understanding the long-term impact of Tele-CBT on both psychological and weight outcomes will provide valuable insights into the optimization of post-surgical care for MBS patients, particularly in addressing the psychological factors that contribute to recurrent weight gain.

## Methods

Ethics approval was obtained from all three hospital-based MBS surgery programs that participants were recruited from. This RCT was registered with ClinicalTrials.gov (ID: NCT03315247). This manuscript used the Consolidated Standards of Reporting Trials (CONSORT) reporting guidelines [18].

### Study Design

**Fig. 1:**
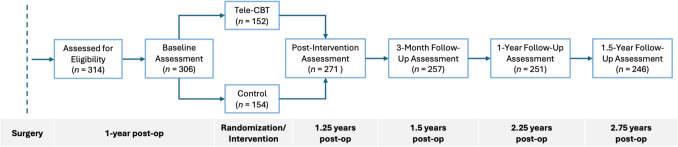
Study design

The study design was a two-arm longitudinal RCT (Fig. 1) and used a parallel group design with a 1:1 allocation ratio. One arm received treatment as usual/standard MBS care and the other received Tele-CBT. Participants completed study measures at five time points: baseline (1-year post-MBS), immediately post-Tele-CBT, 3-month, 12-month, and 18-month follow-up.

### Participants and Recruitment

Participants were recruited from three Bariatric Centres of Excellence (BCoE) in Ontario, Canada (Toronto Western Hospital, Humber River Hospital, and The Ottawa Hospital) between February 2018 and December 2021. BCoE clinicians informed patients approaching 12 months post-surgery about the study and referred those who expressed interest to the research team. Once connected, the research coordinator provided detailed study information, screened for eligibility, and obtained written informed consent. Participants did not need to have symptoms of psychological distress or pathological eating to be eligible to participate. To be eligible to enrol in the BCoE and be considered for MBS, patients needed to have a BMI of $\ge$ 40 kg/m^2^ or a BMI of $\ge$ 35 kg/m^2^ with at least one obesity-related comorbidity, be 18 years of age or older, and meet all BCoE requirements (e.g., abstinence from cigarette smoking). Participants were eligible to participate in the current RCT if they were 1-year post-MBS, were fluent in English, and had access to a telephone for Tele-CBT sessions and an Internet connection to complete online surveys. Participants were ineligible if they had active suicidal ideation or poorly controlled psychiatric illness that would make it difficult to participate in Tele-CBT (e.g., current psychosis). Recruitment was completed in December 2021, as no further recruitment was required to achieve adequate power for the planned analyses.

### Study Procedures

Participants first completed the baseline questionnaire packet and a demographic form. Participants were subsequently randomized to either the Tele-CBT or Control condition using open randomization. The permuted block technique was used with block sizes of 4 and 6, which were stratified by BCoE site [19]. The randomization sequence was generated using R statistical software version 3.4.3. The random allocation sequence was developed by a biostatistician (CM) and enrollment and participant assignment was conducted by the research coordinator (SL).

Participants assigned to the standard care control group attended routine clinic visits at their respective BCoE site. During these visits, patients typically receive education on MBS aftercare, such as nutrition education, from members of the multidisciplinary team. At 1-year post-MBS, patients typically meet with a surgeon, nurse/nurse practitioner, dietitian, and at least one psychosocial team member (i.e., social worker, psychologist) with additional post-op appointments with a social worker, psychologist, or psychiatrist as needed. This psychosocial check-in is part of standard care but is not considered a form of structured psychological therapy. While patients may request individual sessions with a mental health clinician (e.g., clinical psychologist, social worker) following surgery, these services are limited in availability and often associated with substantial wait times (typically 6 months to 1 year). Some sites also offer group-based supports such as a body image group or mindful eating program. Although these groups are not formal CBT interventions, some incorporate CBT-informed strategies. Physician or nurse/nurse practitioner follow-ups also occurred at 2 and 3 years post-MBS.

### Intervention

The intervention entailed 6 weekly 1-h sessions, followed by a seventh 1-h “booster” session delivered 1 month later. The protocol has been previously described in the literature and is summarized in Appendix 1 [15]. Five clinical psychology doctoral students who had experience working with MBS patients and received training on the protocol delivered the treatment. They were supervised by a registered clinical psychologist (SC) and attended bi-weekly group supervision meetings and individual supervision meetings as needed.

### Study Measures

#### Primary Outcome: Post-operative Percentage of Total Weight Loss (%TWL)

Participants self-reported their weight in either pounds or kilograms and emailed an image of their scale to the research coordinator to increase the reliability of self-reporting. Percentage of total weight loss (%TWL) was calculated using the formula = ([baseline weight in kilograms − postintervention or follow-up weight in kilograms]/baseline weight in kilograms) × 100. Typically, in the literature, %TWL is calculated using the patients’ pre-surgical weight as the baseline [20]. However, to isolate the effects of the Tele-CBT intervention, baseline weight was measured at the start of the study, which occurred at 1-year post-MBS.

#### Secondary Outcome: Disordered Eating

The Binge Eating Scale (BES) [21] is a 16-item self-report scale used to assess cognitive and behavioral symptoms of binge eating, which may be indicative of an eating disorder. The scale was specifically designed for individuals with obesity and has previously been validated in MBS populations [22]. Scores on the BES range from 0 to 46, with moderate and severe levels of binge eating corresponding to cutoff scores of 17 and 27, respectively, although a recent study has recommended a BES cut-off score of ≥ 15 in MBS patients [23]. In the current study, internal consistency at baseline was excellent (Cronbach’s α = 0.90).

The Emotional Eating Scale (EES) [24] is a 25-item self-report measure where respondents rate their urge to eat in response to 25 negative emotions. Items are rated on a Likert scale ranging from 1 (*no desire to eat*) to 5 (*overwhelming urge to eat*). Internal consistency at baseline for this scale was excellent overall (Cronbach’s α for total EES scale = 0.96). Both the EES and BES have been used to assess disordered eating in patients undergoing MBS [16].

#### Secondary Outcome: Psychological Distress

The Patient Health Questionnaire (PHQ-9) [25] is a nine-item questionnaire that assesses the presence and severity of depression symptoms over the last 2 weeks. Items are rated on a Likert scale ranging from 0 (*not at all*) to 3 (*nearly every day*) with a maximum total score of 27. In the current study, internal consistency was very good (Cronbach’s α = 0.85).

The Generalized Anxiety Disorder (GAD-7) [26] is a seven-item questionnaire that assesses the presence and severity of worry and anxiety symptoms over the last 2 weeks. Items are rated on a Likert scale ranging from 0 (*not at all*) to 3 (*nearly every day*) with a maximum total score of 21. In the current study, internal consistency was very good (Cronbach’s α = 0.88). Psychological distress has been previously measured in MBS patient populations using the PHQ-9 and GAD-7 [16].

### Sample Size and Power

Based on literature and consultations with surgeons in the BCoE, a 5% weight loss at 1-year post-MBS was deemed to be clinically meaningful given impact on obesity-related outcomes [27] and past CBT trials (7.5 to 10% TWL) [28, 29]. Our power calculation, detailed in Appendix 2, determined that 124 participants per group were needed to detect a clinically significant difference with 80% power and a type I error rate of 5%. Accounting for 30% attrition, our target was to enroll 175 participants per group.

### Statistical Analysis

Statistical analyses were performed using SPSS Statistics version 25.0. Shapiro–Wilk tests were used to determine whether continuous outcome variables were normally distributed. All outcomes were non-normally distributed and were log(*x* + 1) transformed for analyses. %TWL was log(*x* + 50) transformed for analysis to account for negative values. Linear mixed models (LMM) were used to evaluate treatment effects, as this technique can account for the hierarchical data structure, including within-participant repeated measures and between-group data. LMM with random intercepts were generated for each outcome variable, incorporating fixed effects for group (Tele-CBT vs. Control), time, and the group-by-time interaction. Pairwise comparisons of estimated marginal means were conducted using Bonferroni-adjusted post hoc tests to examine differences between groups at each time point. For reporting, estimated means and SEs from mixed models were back-transformed to their original units as: exp(mean log) − 1 ± exp(mean log) × (exp(SE log) − 1). %TWL estimated means were back-transformed as exp(mean log – 50) to account for original log transformations.

### Missing Data

Linear mixed models (LMM) are generally robust at handling missing data by leveraging all available data points for each participant and assuming data are missing at random [30]. We included participants with at least one observed outcome in the LMM implementing the intent-to-treat principle and no imputation of missing values.

## Results

### Participant Flow and Characteristics

**Fig. 2:**
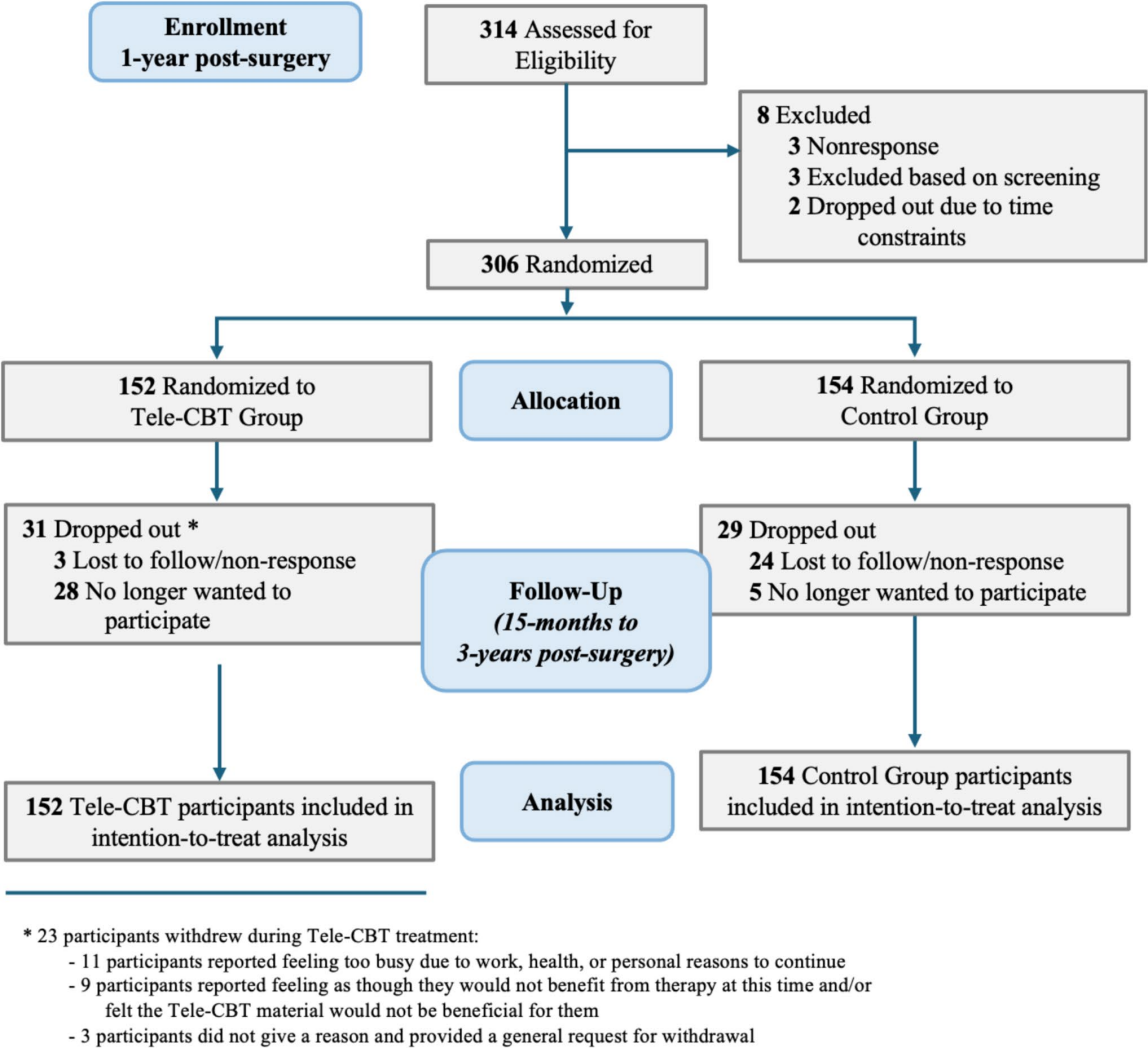
CONSORT diagram of participant flow

Table 1 summarizes the demographic information for both patient groups. Participants ranged in age between 20 and 69 years old. The majority of participants were female (83.3%), were Caucasian (76.5%), were in a relationship (60.8%), completed college or university (64.7%), were employed full-time (69.3%), and earned a household income greater than $80,000 (48.0%). Of the 314 patients who consented to participate, 306 (97%) completed the baseline questionnaires and were randomized to either the Tele-CBT (*n* = 152) or Control (*n* = 154) group. Randomization resulted in generally comparable groups across most baseline sociodemographic variables, with no statistically significant differences in age, sex, race/ethnicity (collapsed into White vs. not White), relationship status, occupational status, education, income, or study site (all *p* > 0.05). However, participants in the Tele-CBT group had significantly higher pre-operative BMI, baseline BMI, and baseline weight compared to the Control group (all *p* < 0.01; see Table 1). Sixty participants dropped out, with reasons for withdrawal detailed in the CONSORT participant flow diagram (Fig. 2).

**Table 1:** Participant characteristics

Characteristic	Participants, no. (%)			
	Tele-CBT (***n*** = 152)	Control (***n*** = 154)	Overall (***n*** = 306)	***p***-value^b^
Study site				0.960
Toronto Western Hospital	130 (85.5)	131 (85.1)	261 (85.3)	
Humber River Hospital	17 (11.2)	17 (11.0)	34 (11.1)	
The Ottawa Hospital	5 (3.3)	6 (3.9)	11 (3.6)	
Age at baseline^a^ (years), *n* = 305	46.86 (10.33)	48.23 (9.61)	47.55 (9.98)	0.232
Weight at baseline^a^ (kg), *n* = 305	98.23 (26.28)	89.35 (19.56)	93.78 (23.5)	0.001*
BMI				
Pre-operative	48.50 (7.28)	45.56 (6.90)	47.02 (7.23)	< 0.001*
At baseline^a^, *n* = 303	34.77 (8.46)	32.20 (6.06)	33.48 (7.46)	0.003*
1.25 years post-op, *n* = 268	34.23 (7.72)	31.91 (6.22)	32.98 (7.04)	
1.50 years post-op, *n* = 254	34.32 (8.05)	31.85 (6.53)	33.03 (7.40)	
2.25 years post-op, *n* = 246	35.00 (8.20)	32.50 (6.70)	33.72 (7.56)	
2.75 years post-op, *n* = 239	36.42 (8.28)	33.57 (6.80)	34.97 (7.68)	
Surgery type (Roux-en-Y)	117 (77%)	131 (85.1%)	248 (81%)	
Sex (female)	126 (82.9%)	129 (83.8%)	255 (83.3%)	0.739
Race/ethnicity				0.618
Arab/West Asian	3 (2%)	7 (4.5%)	10 (3.3%)	
Black	12 (7.9%)	11 (7.1%)	23 (7.5%)	
Indigenous	2 (1.3%)	1 (0.6%)	3 (1%)	
Latin American	4 (2.6%)	5 (3.2%)	9 (2.9%)	
South Asian	2 (1.3%)	1 (0.6%)	3 (1%)	
South East Asian	2 (1.3%)	1 (0.6%)	3 (1%)	
White (Caucasian)	114 (75%)	120 (77.9%)	234 (76.5%)	
Other	12 (7.9%)	7 (4.5%)	19 (6.2%)	
Missing	1 (0.7%)	0 (0%)	1 (0.3%)	
Relationship status				0.333
Married/common-law	89 (58.6%)	97 (63%)	186 (60.8%)	
Divorced/separated	22 (14.5%)	20 (13%)	42 (13.7%)	
Single	37 (24.3%)	37 (24%)	74 (24.2%)	
Widowed	3 (2%)	0 (0%)	3 (1%)	
Missing	1 (0.7%)	0 (0%)	1 (0.3%)	
Occupational status				0.447
Full-time	98 (64.5%)	114 (74%)	212 (69.3%)	
Part-time	12 (7.9%)	10 (6.5%)	22 (7.2%)	
Retired	14 (9.2%)	12 (7.8%)	26 (8.5%)	
Social assistance	1 (0.7%)	1 (0.6%)	2 (0.7%)	
Disability	17 (11.2%)	8 (5.2%)	25 (8.2%)	
Unemployed	9 (5.9%)	9 (5.8%)	18 (5.9%)	
Missing	1 (0.7%)	0 (0%)	1 (0.3%)	
Education				0.079
Some high school	7 (4.6%)	1 (0.6%)	8 (2.6%)	
High school graduate	12 (7.9%)	15 (9.7%)	27 (8.8%)	
Some college/university	36 (23.7%)	34 (22.1%)	70 (22.9%)	
College or university graduate	94 (61.8%)	104 (67.5%)	198 (64.7%)	
Missing	3 (2%)	0 (0%)	3 (1%)	
Household income				0.260
$0–$30,000	23 (15.1%)	15 (9.7%)	38 (12.4%)	
$30,000–$59,999	26 (17.1%)	38 (24.7%)	64 (20.9%)	
$60,000–$79,999	27 (17.8%)	26 (16.9%)	53 (17.3%)	
$80,000 +	75 (49.3%)	72 (46.8%)	147 (48.0%)	
Missing	1 (0.7%)	3 (1.9%)	4 (1.3%)	

*BMI* body mass index (calculated as weight in kilograms divided by height in meters squared)

^a^Baseline refers to when participants were recruited to participate in the study and were 1-year post-MBS

^b^*p*-values reflect group comparisons between Tele-CBT and Control conditions. Independent *t*-tests were used for continuous variables, and chi-square tests were used for categorical variables

*Indicates statistical significance at *p* < 0.05

### Comparison of Tele-CBT and Control Groups on Outcomes Across Time

The LMM analysis revealed significant interactions between treatment group and time for several outcomes, indicating differential changes over time between the Tele-CBT and Control groups. There were significant group-by-time interactions for the BES (*F*_4,663.38_ = 8.99, *p* = < 0.001), EES (*F*_4,1018.37_ = 6.15, *p* < 0.001), PHQ (*F*_4, 1022.60_ = 10.87, *p* < 0.001), and GAD (*F*_4__,1030.61_ = 10.19, *p* < 0.001). However, there were no statistically significant differences between the groups at any time point post-treatment for weight loss (%TWL), suggesting similar weight trajectories across groups (*F*_3,719.06_ = 1.40, *p* = 0.24). There was a significant effect of time on %TWL (F_3, 719.06_) = 66.35, *p* < 0.001, indicating that participants in both groups experienced less %TWL over time. No significant harms or unintended effects were observed in either group during the study.

See Table 2 for pairwise comparisons of estimated marginal means (EMMs) between groups across all time points for outcome variables. For disordered eating, the Tele-CBT group showed significant reductions in BES scores at post-intervention (*p* < 0.001) and 3-month follow-up (*p* = 0.04), but these effects were not sustained at 12- or 18-month follow-up. EES scores significantly decreased in the Tele-CBT group at post-intervention (*p* = 0.01), 3-month (*p* = 0.03), and 12-month follow-up (*p* = 0.01), though not at 18-month follow-up. For psychological distress, the Tele-CBT group reported significantly lower PHQ-9 scores at post-intervention (*p* < 0.001), and 3-month follow-up (*p* < 0.001), and lower GAD-7 scores at post-intervention (*p* < 0.001) and 3-month follow-up (*p* < 0.01). However, the improvements in psychological distress were not sustained at 12- or 18-month follow-up.

**Table 2:** Pairwise comparisons of estimated marginal means (EMMs) between Tele-CBT and control groups across all time points for outcome variables

Outcome variables	Time	Tele-CBT group EMM (SE)^a^	Control group EMM (SE)^a^	Mean difference	SE	*p*-value^b^	95% CI
BES	Baseline^c^	9.87 (0.80)	10.40 (0.83)	− 0.03	0.10	0.73	− 0.23, 0.16
	Post-intervention	**5.33 (0.49)**	**9.38 (0.77)**	− **0.46**	**0.11**	< 0.001**	− **0.67,** − **0.25**
	3-month F/U	**6.47 (0.58)**	**9.10 (0.76)**	− **0.23**	**0.11**	0.04*	− **0.44,** − **0.01**
	12-month F/U	7.31 (0.79)	10.43 (1.04)	− 0.18	0.17	0.27	− 0.51, 0.14
	18-month F/U	6.79 (1.47)	9.39 (1.65)	− 0.64	0.38	0.09	− 1.39, 0.10
EES	Baseline^c^	46.78 (1.79)	45.27 (1.72)	0.03	0.05	0.52	− 0.07, 0.13
	Post-intervention	**37.70 (1.53)**	**44.17 (1.71)**	− **0.14**	**0.05**	0.01*	− **0.25,** − **0.04**
	3-month F/U	**39.70 (1.62)**	**45.19 (1.79)**	− **0.12**	**0.06**	0.03*	− **0.23,** − **0.01**
	12-month F/U	**42.77 (1.74)**	**50.38 (2.01)**	− **0.15**	**0.06**	0.01*	− **0.26,** − **0.04**
	18-month F/U	45.82 (1.88)	50.16 (2.01)	− 0.09	0.06	0.10	− 0.20, 0.02
PHQ-9	Baseline^c^	3.94 (0.37)	4.15 (0.39)	− 0.04	0.10	0.68	− 0.24, 0.16
	Post-intervention	**2.22 (0.26)**	**4.30 (0.41)**	− **0.47**	**0.11**	< 0.001**	− **0.68,** − **0.25**
	3-month F/U	**2.67 (0.29)**	**4.66 (0.44)**	− **0.39**	**0.11**	< 0.001**	− **0.61,** − **0.17**
	12-month F/U	3.41 (0.35)	4.63 (0.44)	− 0.2	0.11	0.07	− 0.42, 0.02
	18-month F/U	4.11 (0.41)	4.72 (0.45)	− 0.09	0.12	0.42	− 0.32, 0.13
GAD-7	Baseline^c^	3.37 (0.34)	3.19 (0.32)	0.04	0.10	0.68	− 0.16, 0.25
	Post-intervention	**1.84 (0.23)**	**3.34 (0.34)**	− **0.40**	**0.11**	< 0.001**	− **0.62,** − **0.18**
	3-month F/U	**2.02 (0.25)**	**3.46 (0.36)**	− **0.35**	**0.11**	< 0.01*	− **0.57,** − **0.13**
	12-month F/U	2.58 (0.29)	3.31 (0.35)	− 0.14	0.12	0.21	− 0.37, 0.08
	18-month F/U	3.23 (0.35)	3.00 (0.33)	0.09	0.12	0.45	− 0.14, 0.32
%TWL	Baseline^c^	n/a	n/a				
	Post-intervention	1.25 (0.87)	0.99 (0.80)	− 0.01	0.02	0.82	− 0.04, 0.05
	3-month F/U	0.68 (0.86)	0.60 (0.82)	< 0.01	0.02	0.97	− 0.05, 0.05
	12-month F/U	− 3.07 (0.81)	− 1.90 (0.79)	− 0.03	0.02	0.29	− 0.07, 0.02
	18-month F/U	− 7.76 (0.78)	− 5.97 (0.80)	− 0.05	0.03	0.07	− 0.10, < 0.01

*BES* binge eating scale, *EES* emotional eating scale, *PHQ-9* patient health questionnaire 9-item, *GAD-7* generalized anxiety disorder 7-item, %*TWL* percentage of total weight loss, using the formula = ([baseline weight in kilograms − postintervention or follow-up weight in kilograms]/baseline weight in kilograms) × 100

^a^Estimated marginal means (EMMs) and standard errors (SE) were derived from the linear mixed models (LMM) and have been back-transformed for interpretability

^b^Bonferroni adjustment has been implemented for multiple comparisons

^c^Baseline refers to when participants were recruited to participate in the study and were 1-year post-MBS

*sig at *p* < 0.05

**sig at *p* < 0.001

## Discussion

The current study sought to examine whether the psychological benefits of a brief Tele-CBT intervention persist over time and contribute to better weight outcomes among post-MBS patients. Participants were recruited 1 year following MBS, a period when patients typically report maximum weight loss and improvements in disordered eating and psychological functioning [5]. The benefits to emotional eating were sustained at 12-month follow-up, whereas benefits waned after 3 months for binge eating, depression, and anxiety. Although the improvements on these measures were no longer statistically significant, the mean scores continued to fall in the “minimal” range across the 18-month follow-up period.

The Tele-CBT intervention intended to sustain the psychological benefits following surgery and mitigate recurrent weight gain. Although Tele-CBT focuses on improving eating behaviors, it is not a weight loss intervention per se. Many patients set goals, such as increasing the variety of food consumed and eating at regular intervals, that may not impact their daily caloric intake but may reduce vulnerability to emotional or binge eating. Despite the significant benefits of Tele-CBT for disordered eating and psychological functioning in comparison to standard care, the %TWL trajectories were comparable in both groups. Both groups in the current study experienced a small amount of recurrent weight gain during the follow-up period (extending approximately 3-years post-surgery). %TWL from the pre-operative assessment (rather than baseline) are presented in Appendix 3. The overall trajectory was consistent with prior research, [5, 6] demonstrating a gradual decline in %TWL over time after the first year post-surgery.

The limited durability of intervention effects observed at 18-month follow-up, approximately 2.5 to 3 years post-surgery, should be interpreted in light of the intervention’s scope and intensity. Tele-CBT was brief, consisting of only seven sessions delivered over a few months (7 h total), and was not designed as a long-term therapeutic approach. Given that obesity is a chronic, progressive disease [31] and that the postoperative period beyond 2 years is associated with increased risk for weight regain and disordered eating recurrence, [32] these findings underscore the need for more sustained psychosocial support. Rather than suggesting that CBT is ineffective in this context, the results point to the limitations of time-limited interventions and highlight the potential value of booster sessions, or ongoing access to CBT-informed support as part of routine post-bariatric care.

While total weight loss (%TWL) was the designated primary outcome, it does not fully reflect the clinical value of the Tele-CBT intervention. Consistent with recommendations from the International Consortium for Health Outcomes Measurement, [33] which highlights the importance of patient-centered outcomes in obesity care (such as psychological well-being, social functioning, and dietary behaviors), our findings demonstrate that Tele-CBT effectively targets disordered eating and psychological distress, which are factors associated with health-related quality of life for MBS patients [11]. Future research and clinical evaluations should prioritize such multidimensional outcomes to better align with the lived experiences and treatment goals of bariatric surgery patients.

Study limitations include the reliance on self-report outcome measures and the lack of measures assessing other outcomes that matter to patients living with obesity, such as nutritional status and cardiometabolic risk factors [33] that could potentially be impacted by dietary and lifestyle changes made during Tele-CBT. In addition, all programs involved in this multisite RCT offered behavioral support and multidisciplinary team-based care as part of standard care which may limit generalizability to MBS patients with less intensive follow-up care and reduce the observed impact of Tele-CBT in comparison to a no-treatment Control group. Perhaps due to the intensive multidisciplinary post-operative follow-up, participants reported low mean scores on measures of disordered eating and psychological distress at the time of recruitment. Another multisite RCT is underway to examine whether Tele-CBT has a more robust effect when patients experiencing higher levels of psychological distress and disordered eating symptoms are targeted for recruitment. Another limitation of our study is that we did not systematically assess psychological treatment received during the study period. Participants in both the Control and Tele-CBT groups were not restricted from seeking additional support, and while randomization should have balanced this across groups, we cannot rule out its potential influence on outcomes.

Study strengths include the multisite RCT design, large sample size, respectable rate of treatment completion, outcome measures with strong psychometric properties, and respectable rate of retention across multiple follow-up periods. The high retention rate with Tele-CBT suggests that Tele-CBT is acceptable as a virtually delivered intervention which is important considering challenges with travelling to bariatric centres.

## Conclusion

Standard post-operative follow-up decreases in intensity over time and yet some benefits of Tele-CBT were maintained at 2 years post-MBS, suggesting that Tele-CBT may serve a useful role in optimizing post-operative care for MBS patients. Considering that dietary behaviors and mental health are among the outcomes that matter most to patients living with obesity, [33] additional research is warranted to determine the optimal timing and number of Tele-CBT booster sessions required to sustain those effects. Obesity is a chronic, multi-system disease resulting from complex interactions between genetic, environmental, social, and psychological factors that requires ongoing medical and psychosocial support [34].

## Supplementary Information

Below is the link to the electronic supplementary material.

Supplementary Material 1 (DOCX 17.9 KB)

## Data Availability

Data Availability Statement: The anonymized data that support the findings of this study are available from the corresponding author upon request.

## References

[1] Adams TD, Davidson LE, Litwin SE, et al. Weight and metabolic outcomes 12 years after gastric bypass. N Engl J Med. 2017;377(12):1143–55. 28930514 10.1056/NEJMoa1700459 PMC5737957

[2] Eisenberg D, Shikora SA, Aarts E, et al. 2022 American Society for Metabolic and Bariatric Surgery (ASMBS) and International Federation for the Surgery of Obesity and Metabolic Disorders (IFSO): indications for metabolic and bariatric surgery. Surg Obes Relat Dis. 2022;18(12):1345–56. 36280539 10.1016/j.soard.2022.08.013

[3] Syn NL, Cummings DE, Wang LZ, et al. Association of metabolic-bariatric surgery with long-term survival in adults with and without diabetes: a one-stage meta-analysis of matched cohort and prospective controlled studies with 174 772 participants. Lancet. 2021;397(10287):1830–41. 33965067 10.1016/S0140-6736(21)00591-2

[4] Youssef A, Keown-Stoneman C, Maunder R, et al. Differences in physical and mental health-related quality of life outcomes 3 years after bariatric surgery: a group-based trajectory analysis. Surg Obes Relat Dis. 2020;16(11):1837–49. 32737009 10.1016/j.soard.2020.06.014

[5] Courcoulas AP, King WC, Belle SH, et al. Seven-year weight trajectories and health outcomes in the longitudinal assessment of bariatric surgery (LABS) study. JAMA Surg. 2018;153(5):427–34. 29214306 10.1001/jamasurg.2017.5025 PMC6584318

[6] King WC, Hinerman AS, Belle SH, et al. Comparison of the performance of common measures of weight regain after bariatric surgery for association with clinical outcomes. JAMA. 2018;320(15):1560–9. 30326125 10.1001/jama.2018.14433 PMC6233795

[7] Zefreh H, Amani-Beni R, Sheikhbahaei E, et al. What about my weight? Insufficient weight loss or weight regain after bariatric metabolic surgery. Int J Endocrinol Metab. 2023;21(4): e136329. 38666043 10.5812/ijem-136329 PMC11041817

[8] Mauro M, Papelbaum M, Brasil MAA, et al. Is weight regain after bariatric surgery associated with psychiatric comorbidity? A systematic review and meta-analysis. Obes Rev. 2019;20(10):1413–25. 31322316 10.1111/obr.12907

[9] Nasirzadeh Y, Kantarovich K, Wnuk S, et al. Binge eating, loss of control over eating, emotional eating, and night eating after bariatric surgery: results from the Toronto Bari-PSYCH cohort study. Obes Surg. 2018;28(7):2032–9. 29411241 10.1007/s11695-018-3137-8

[10] Alyahya RA, Alnujaidi MA. Prevalence and outcomes of depression after bariatric surgery: a systematic review and meta-analysis. Cureus. 2022;14(6): e25651. 35784972 10.7759/cureus.25651 PMC9249077

[11] Devlin MJ, King WC, Kalarchian MA, et al. Eating pathology and experience and weight loss in a prospective study of bariatric surgery patients: 3-year follow-up. Int J Eat Disord. 2016;49(12):1058–67. 27425771 10.1002/eat.22578 PMC5161707

[12] David LA, Sijercic I, Cassin SE. Preoperative and post-operative psychosocial interventions for bariatric surgery patients: a systematic review. Obes Rev. 2020;21(4): e12926. 31970925 10.1111/obr.12926

[13] Cassin SE, Sockalingam S, Du C, et al. A pilot randomized controlled trial of telephone-based cognitive behavioural therapy for preoperative bariatric surgery patients. Behav Res Ther. 2016;80: 17–22. 10.1016/j.brat.2016.03.001 PMC5468091 26990279

[14] Sockalingam S, Leung SE, Hawa R, et al. Telephone-based cognitive behavioural therapy for female patients 1-year post-bariatric surgery: a pilot study. Obes Res Clin Pract. 2019;13(5): 499–504. 10.1016/j.orcp.2019.07.003 31409544

[15] Sockalingam S, Leung SE, Ma C, et al. Efficacy of telephone-based cognitive behavioral therapy for weight loss, disordered eating, and psychological distress after bariatric surgery: a randomized clinical trial. JAMA Netw Open. 2023;6(8): e2327099. 10.1001/jamanetworkopen.2023.27099 PMC10401302 37535357

[16] Sockalingam S, Cassin SE, Wnuk S, et al. A pilot study on telephone cognitive behavioral therapy for patients six-months post-bariatric surgery. Obes Surg. 2017;27(3): 670–5. 10.1007/s11695-016-2322-x PMC5468092 27491293

[17] Santiago VA, Cassin SE, Wnuk S, et al. “If you’re offered help, take it”: a qualitative study examining bariatric patients’ experience of telephone-based cognitive behavioural therapy. Clin Obes. 2021;11(2): e12431. 33251753 10.1111/cob.12431

[18] Schulz KF, Altman DG, Moher D, CONSORT Group. CONSORT 2010 statement: updated guidelines for reporting parallel group randomised trials. BMJ. 2010;340: c332. 10.1136/bmj.c332R. 10.1136/bmj.c332 PMC2844940 20332509

[19] Broglio K. Randomization in clinical trials: permuted blocks and stratification. In: Livingston EH, Lewis RJ, editors. JAMA guide to statistics and methods. New York: McGraw-Hill Education; 2019. Available from: https://jamaevidence.mhmedical.com/content.aspx?bookid=2742§ionid=233567426. Accessed 8 Jan 2025. 10.1001/jama.2018.6360 29872845

[20] van Rijswijk AS, van Olst N, Schats W, et al. What is weight loss after bariatric surgery expressed in percentage total weight loss (%TWL)? A systematic review. Obes Surg. 2021;31(8):3833–47. 34002289 10.1007/s11695-021-05394-x

[21] Gormally RC, Black MS, Daston LM, et al. The assessment of binge eating severity among obese persons. Addict Behav. 1982;7(1):47–55. 7080884 10.1016/0306-4603(82)90024-7

[22] Grupski AE, Hood MM, Hall BJ, et al. Examining the binge eating scale in screening for binge eating disorder in bariatric surgery candidates. Obes Surg. 2013;23(1):1–6. 23104387 10.1007/s11695-011-0537-4 PMC4874644

[23] Jeong H, Hapenciuc G, Meza E, et al. Reevaluating the binge eating scale cut-off using DSM-5 criteria: analysis and replication in preoperative metabolic and bariatric surgery samples. Surg Obes Relat Dis. 2023;19(9):945–9. 36959027 10.1016/j.soard.2023.02.014

[24] Arnow B, Kenardy J, Agras WS. The emotional eating scale: the development of a measure to assess coping with negative affect by eating. Int J Eat Disord. 1995;18(1):79–90. 7670446 10.1002/1098-108x(199507)18:1<79::aid-eat2260180109>3.0.co;2-v

[25] Kroenke K, Spitzer RL, Williams JB. The PHQ-9: validity of a brief depression severity measure. J Gen Intern Med. 2001;16(9):606–13. 11556941 10.1046/j.1525-1497.2001.016009606.x PMC1495268

[26] Spitzer RL, Kroenke K, Williams JB, et al. A brief measure for assessing generalized anxiety disorder: the GAD-7. Arch Intern Med. 2006;166(10):1092–7. 16717171 10.1001/archinte.166.10.1092

[27] Yamakage H, Jo T, Tanaka S, et al. Five percent weight loss is a significant 1-year predictor and an optimal 5-year cut-off for reducing the number of obesity-related cardiovascular disease risk components: The Japan Obesity and Metabolic Syndrome Study. Front Endocrinol (Lausanne). 2024;14:1343153. 10.3389/fendo.2024.1343153 PMC11005029 38601201

[28] Cooper Z, Doll HA, Hawker DM, et al. Testing a new cognitive behavioural treatment for obesity: a randomized controlled trial with three-year follow-up. Behav Res Ther. 2010;48(8):706–13. 10.1016/j.brat.2010.03.008. 20691328 10.1016/j.brat.2010.03.008 PMC2923743

[29] Werrij MQ, Jansen A, Mulkens S, et al. Adding cognitive therapy to dietetic treatment is associated with less relapse in obesity. J Psychosom Res. 2009;67(4):315–24. 10.1016/j.jpsychores.2008.12.011. 19773024 10.1016/j.jpsychores.2008.12.011

[30] Diggle PJ, Heagerty PJ, Liang KY, et al. Analysis of longitudinal data. 2nd ed. Oxford: Oxford University Press; 2013. https://global.oup.com/academic/product/analysis-oflongitudinal-data-9780198524847?cc=ca&lang=en&.

[31] Mechanick JI, Hurley DL, Garvey WT. Adiposity-based chronic disease as a new diagnostic term: the American Association of Clinical Endocrinologists and American College of Endocrinology position statement. Endocr Pract. 2017;23(3):372–8. 27967229 10.4158/EP161688.PS

[32] Noria SF, Shelby RD, Atkins KD, et al. Weight regain after bariatric surgery: scope of the problem, causes, prevention, and treatment. Curr Diab Rep. 2023;23(3):31–42. 36752995 10.1007/s11892-023-01498-z PMC9906605

[33] International Consortium for Health Outcomes Measurement (ICHOM). Adult obesity: patient-centered outcome measure [Internet]. Cambridge (MA): ICHOM; [cited 2025 Feb 21]. Available from: https://www.ichom.org/patient-centered-outcome-measure/adult-obesity/. Accessed 21 Feb 2025.

[34] Sharma AM. M, M, M & M: a mnemonic for assessing obesity. Obes Rev. 2010;11(11):808–9. 21182728 10.1111/j.1467-789X.2010.00766.x

