# Supplementary material for: A Randomized-Controlled Trial Examining Telephone-Based Cognitive Behavioral Therapy for Patients After Metabolic and Bariatric Surgery: 18 Month Follow-up Results
Source: Obes Surg. 2025 Sep 1;35(10):4103–13. doi: 10.1007/s11695-025-08163-2 (PMC12540552; doi:10.1007/s11695-025-08163-2)
Supplement: Supplementary file 1 — Supplementary Material 1 (DOCX 17.9 KB) [file 11695_2025_8163_MOESM1_ESM.docx]

**Appendix 1.** Description of Tele-CBT Intervention

The Tele-CBT intervention entailed 6 weekly 1-hour sessions, followed by a seventh 1-hour “booster” session delivered one month later. Five clinical psychology doctoral students who had experience working with MBS patients and received training on the protocol delivered the treatment. They were supervised by a registered clinical psychologist (SC) and attended bi-weekly group supervision meetings and individual supervision meetings as needed.

The sessions focused on treatment goal setting, scheduling regular meals and snacks, planning pleasurable activities as an alternative to overeating, reducing vulnerability to overeating, engaging in self-care, discussing body image, and sustaining long-term lifestyle changes. Participants were encouraged to complete worksheets between sessions (e.g., food records, thought records) and put skills learned into practice (e.g., engaging in pleasurable activities). In the final booster session, participants were able to troubleshoot any emergent challenges they experienced in implementing the skills and develop a relapse prevention plan. Patients could choose where to conduct Tele-CBT sessions, but they were encouraged to complete study components in a quiet and private location where they could easily access the Tele-CBT materials.

| **Session #** | **Topic** | **Brief Description** |
| --- | --- | --- |
| **1** | **Overview of CBT and Goal Setting** | Discuss the rationale for using cognitive behavioural therapy (CBT), understand different types of overeating, and discuss treatment goals.  *Homework:* Complete the worksheet ‘My Cognitive Behavioural Model of Overeating’ to help figure out some reasons you were overeating in the past |
| **2** | **Self-Monitoring** | Understand why it’s important to start and maintain regular patterns of eating *Homework:* Keep a food record |
| **3** | **Alternate Activities and Self-Care** | Understand the importance of self-care and plan other pleasurable activities that don’t involve food and eating  *Homework:* Choose 2 pleasurable activities to do this week |
| **4** | **Challenging Eating Situations & Changing Problem Thoughts** | Learn ways to problem-solve so that you can handle challenging eating situations  *Homework:* Complete handout on handling challenging eating situations and CBT thought records |
| **5** | **Problem-Solving & Body Image** | Combine problem-solving and working through your problem thoughts so that you do not overeat or engage in other unhealthy behaviours. Understand contributions to your body image.  *Homework:* Complete worksheets on problem-solving (e.g., situational analysis method), body checking behaviours, and the body image journal |
| **6** | **Keep on Track after Your Surgery** | Revisit progress towards treatment goals and think about long-term lifestyle changes after surgery  *Homework*: Complete ambivalence worksheet |
| **7** | **Relapse Prevention** | Reflect on the progress you have made towards your treatment goals over the past month. Troubleshoot any issues that arose. Develop a relapse prevention plan to help you maintain the improvements you have made.  *Homework:* Continue setting goals for yourself and monitoring your progress towards them |

**Appendix 2.**

**Sample Size and Power**

Based on literature and consultations with surgeons in the BCoE, a 5% weight loss at one-year post-MBS was deemed to be clinically meaningful given impact on obesity-related outcomes^30^ and past CBT trials (7.5% to 10% TWL).^(31,32)^ We referenced data from Toronto Western Hospital’s Bariatric Program and found that 191 patients showed an average weight of 92 kg at two years post-MBS. The Pearson correlation between weights at 1- and 2- years was 0.8, with a between-patient SD of 21 kg at both time points. A clinically significant 5% weight change equates to 4.5 kg. Assuming a type I error rate of 5%, detecting a 4.5 kg difference between groups at two years requires 124 participants per group to achieve 80% power in an analysis of covariance adjusting for 1-year weight. Accounting for 30% attrition, our target was to enroll 175 participants per group.

**Appendix 3.** Percentage of Total Weight Loss (%TWL) from Pre-Operative Weight (vs. Baseline at 1-Year Post-Op)

|  | **1-year**  **post-op** | **1.25 years post-op** | **1.5- years post-op** | **2.25 years post-op** | **2.75 years post-op** |
| --- | --- | --- | --- | --- | --- |
| **%TWL M(SD)** | 27.97 (11.85) | 28.85 (11.15) | 28.68 (11.90) | 27.17 (12.28) | 24.65(12.17) |
| **n** | 303 | 268 | 254 | 246 | 239 |
